# Mesenchymal stem cell-based Smad7 gene therapy for experimental liver cirrhosis

**DOI:** 10.1186/s13287-020-01911-4

**Published:** 2020-09-14

**Authors:** Dong-Na Su, Shi-Pin Wu, Shang-Zhong Xu

**Affiliations:** 1 Department of Infectious Diseases, Shenzhen People’s Hospital (The Second Clinical Medical College, Jinan University; The First Affiliated Hospital, Southern University of Science and Technology), 1017 Dong Men Bei Road, Luo Hu District, Shenzhen, 518020 Guangdong Province People’s Republic of China; 2 Centre for Atherothrombosis and Metabolic Disease, Hull York Medical School, University of Hull, Hull, HU6 7RX UK

**Keywords:** Mesenchymal stem cells, Smad7, Liver cirrhosis, Fibrosis, TGF-β, Gene therapy

## Abstract

**Background:**

Bone mesenchymal stem cells (MSCs) can promote liver regeneration and inhibit inflammation and hepatic fibrosis. MSCs also can serve as a vehicle for gene therapy. Smad7 is an essential negative regulatory gene in the TGF-β1/Smad signalling pathway. Activation of TGF-β1/Smad signalling accelerates liver inflammation and fibrosis; we therefore hypothesized that MSCs overexpressing the Smad7 gene might be a new cell therapy approach for treating liver fibrosis via the inhibition of TGF-β1/Smad signalling.

**Methods:**

MSCs were isolated from 6-week-old Wistar rats and transduced with the Smad7 gene using a lentivirus vector. Liver cirrhosis was induced by subcutaneous injection of carbon tetrachloride (CCl_4_) for 8 weeks. The rats with established liver cirrhosis were treated with Smad7-MSCs by direct injection of cells into the main lobes of the liver. The expression of Smad7, Smad2/3 and fibrosis biomarkers or extracellular matrix proteins and histopathological change were assessed by quantitative PCR, ELISA and Western blotting and staining.

**Results:**

The mRNA and protein level of Smad7 in the recipient liver and serum were increased after treating with Smad-MSCs for 7 and 21 days (*P* < 0.001). The serum levels of collagen I and III and collagenase I and III were significantly (*P* < 0.001) reduced after the treatment with Smad7-MSCs. The mRNA levels of TGF-β1, TGFBR1, α-SMA, TIMP-1, laminin and hyaluronic acid were decreased (*P* < 0.001), while MMP-1 increased (*P* < 0.001). The liver fibrosis score and liver function were significantly alleviated after the cell therapy.

**Conclusions:**

The findings suggest that the MSC therapy with Smad7-MSCs is effective in the treatment of liver fibrosis in the CCl_4_-induced liver cirrhosis model. Inhibition of TGF-β1 signalling pathway by enhancement of Smad-7 expression could be a feasible cell therapy approach to mitigate liver cirrhosis.

## Introduction

Chronic hepatitis B virus (HBV) infection is a global public health issue with an estimated prevalence of 3.5% in 2017 with 257 million people living with chronic HBV infection [1]. There are 93 million people infected with HBV living in China, including 20 million patients with chronic hepatitis B [2]. Approximately 15–40% of the patients with chronic hepatitis B will develop liver cirrhosis, and 4–5% of the patients may progress towards decompensated liver cirrhosis [3, 4]. The 5-year mortality in patients with compensated liver cirrhosis is 14–20% and with decompensated liver cirrhosis as high as 70–86% [1–3]. Liver cirrhosis is a major cause of morbidity and mortality globally and imposes a substantial health burden on many countries [3]. Currently, there are no treatments available to specifically target fibrosis and cirrhosis, and liver transplantation is the only curative option for patients with liver cirrhosis, but unfortunately, there are simply not enough donated livers to meet the demand for it. Therefore, research on non-surgical strategies to prevent the development of liver cirrhosis is in an urgent need.

Cell therapy is an emerging approach for treating liver cirrhosis [5]. Bone marrow mesenchymal stem cells (MSCs) are multipotent stem cells with low immunogenicity, which can differentiate into liver cells. MSCs can promote liver regeneration and suppress liver fibrosis and thus may partially recover liver function and retard the progression of liver cirrhosis [6]. This new approach has been considered as potentially the most effective non-surgical treatment for liver cirrhosis [6, 7]; however, clinical trials using stem cells seem to not be so successful [5, 8–10]. Therefore, development of stem cells carrying a specific gene related to the regulation of fibrosis could be a new direction for cell therapy.

Liver cirrhosis mainly results from excessive deposition of newly generated extracellular matrix (ECM) in response to injury, which involves the activation of hepatic stellate cells (HSCs) [11]. HSCs are a liver-specific type of pericytes located in the subendothelial layer in the space of Disse. They are major fibrogenic precursor cells that transdifferentiate into fibrogenic myofibroblasts in the liver. The transdifferentiation from a quiescent to a proliferative, migratory and fibrogenic phenotype (i.e. myofibroblast) is driven by inflammatory cytokines, which results in a large amount of ECM synthesis and the expression of alpha-smooth muscle actin (α-SMA). The transforming growth factor (TGF)-β is the main fibrogenic cytokine in the stimulation of HSC transdifferentiation [12] and the signalling of epithelial-to-mesenchymal transitions [13]. Activation of TGF-β1 not only promotes extracellular matrix (ECM) formation but also inhibits ECM degradation and thus accelerates the progression of liver fibrosis [12, 14].

SMADs are transducer proteins and signal TGF-β1 activation to the downstream gene transcription [13, 15]. There are eight SMAD proteins in mammals, six of which have effector function (Smad1—Smad5 and Smad8 (also known as Smad9)) and two of which are inhibitory (Smad6 and Smad7) and mediate the negative feedback of the TGF-β1/SMAD signalling pathway [15, 16]. Overexpression of Smad7 inhibits collagen expression and cell proliferation of HSCs [14]. Our previous study has also demonstrated that rat MSCs overexpressing the Smad7 gene were able to inhibit the fibrogenesis of HSCs [17]. To further validate the therapeutic potential of the Smad7 gene, MSCs overexpressing Smad7 will be injected into rats with liver cirrhosis induced by carbon tetrachloride (CCl_4_). This in vivo study on the rat liver cirrhotic model may provide new insights for stem cell-based gene therapy targeting TGF-β1/Smad signalling.

## Materials and methods

### Animals

All animal experiments were performed under the ethical guidelines of the Ethics Committee of The Second Affiliated Hospital of Jinan University (Shenzhen, China). Six-week-old Wistar rats of either sex (160–200 g body weight) were purchased from the Guangdong Medical Science Experiment Center (Guangdong Province, China). Rats were maintained under constant conditions of temperature (25 °C), air humidity (50–70%) and a 12:12-h light dark cycle. All rats had free access to standard chow and tap water throughout the experiments and were allowed 7–10 days of acclimation to the room and manipulation prior to the experiments. The animal work was performed in the Animal Centre at the North Campus of Sun Yat-sen University.

### Experimental cirrhosis model and transplantation of Smad7-EGFP-MSCs

The CCl_4_-induced cirrhosis model was chosen in this study because it is commonly used for liver cirrhosis study with good reproducibility [18]. Rats were randomly divided into four groups: (1) rats injected with 0.9% NaCl as the control group, (2) rats receiving CCl_4_ to induce liver fibrosis, (3) rats receiving CCl_4_ plus the injection of MSCs and (4) rats receiving CCl_4_ plus the injection of Smad7-EGFP-MSCs. Rat liver fibrosis was induced by subcutaneously injecting 40% carbon tetrachloride-rapeseed oil liquor (5 ml/kg as the initial dose, followed by 3 ml/kg, 2 times a week for 8 weeks). Each group contained 20 rats to monitor the pathological and biomarker changes at different time points.

For the transplantation of MSCs or Smad7-EGFP-MSCs, rats were anesthetized by intraperitoneal injection of 3% pentobarbital sodium (70 mg/kg) before making an upper midline abdominal incision to locate the liver. The MSCs or Smad7-EGFP-MSCs were delivered into the liver by direct injection via the surgical incision, and 3–5 × 10^6^ of Smad7-EGFP-MSCs or MSCs at a volume of 0.4 ml were injected into the liver of each animal in the cell treated groups (~ 0.1 ml each for injection into 3–4 liver lobes), and 0.4 ml phosphate-buffered saline (PBS) was injected in the control groups. The incision was sutured after injections, and all the animals were recovered after surgery. The experimental procedures were approved by the Ethics Committee of Shenzhen People’s Hospital.

### Isolation, purification and characterization of MSCs

Six-week-old male Wistar rats were sacrificed, and their femurs were dissected out. MSCs were isolated and purified from the bone marrow using density gradient centrifugation and the marrow adherence method as described previously [17, 19, 20]. Briefly, bone marrow cells were harvested by carefully washing the marrow cavity with PBS. The cells were centrifuged at 400 g for 10 min, resuspended in the culture medium using a 21-gauge syringe and filtered using a 70-μm nylon mesh filter. The nucleated cells were isolated by density gradient centrifugation with Ficoll/Paque (Amersham Pharmacia). The non-adherent cells were discarded after cell culture for 48 h. The MSCs were cultured in Dulbecco’s modified Eagle’s medium (DMEM)/F12 medium (Thermo Fisher Scientific, Waltham, MA, USA) supplemented with 10% foetal bovine serum (FBS) at a density of 1 × 10^6^ cells/ml. The culture medium was replaced every 3 days. Cells were passaged using 0.05% trypsin-EDTA when 80% confluence was reached. The MSC cell markers were characterized at passage 3 using FACS, including CD31, CD34, CD45, CD73, CD105 and CD106.

### Construction of rat Smad7-EGFP-MSCs

The Entry/Gateway® system was employed to construct the lentiviral vectors that expressed enhanced green fluorescent protein (EGFP)-tagged Smad7 protein. The procedure is similar to our previous report [17]. Briefly, total RNA was extracted from rat cerebral cortex, and the full sequence of Smad7 was obtained by RT-PCR with 100% identity to the GenBank accession No. AH008243.2. Upon cleaving the lentiviral vector, pCDH-CMV-MCS-EF1-copEGFP (Shanghai GenePharma Co., Ltd., Shanghai, China) with restriction enzymes *Eco*RI and *Bam*HI (Shanghai GenePharma Co., Ltd.), the Smad7 cDNA tagged with EGFP was inserted into the vector. The rat MSCs at passage 3 were then infected with the lentiviral vector, and the efficiency of induction was evaluated under a fluorescence microscope. The MSCs with stable expression of Smad7-EGFP were sub-cultured for the experiments of liver injection, and MSCs at the same passage number were used as control.

### Histology and fibrosis quantification

The valuation of liver cirrhosis was exclusively based on histological criteria with characteristics of architectural distortion and the formation of regenerative nodules of hepatocytes surrounded by fibrous tissue. After injection with CCl_4_ for 4 and 8 weeks, rats from the control group and the CCl_4_-treated group were sacrificed and the histology of liver cirrhosis was examined using paraffin-embedded sections. Briefly, portions of the left lateral and the median lobes of the liver were placed in 10% neutral buffered formalin and processed for paraffin embedding. Tissue sections were cut at 6-μm thickness using a microtome and stained with haematoxylin-eosin, Sirius red (Direct Red 80, Sigma-Aldrich) or Masson Trichrome (Bio-Optica Milano SpA). The staining was assessed by a trained pathologist in a blinded way to the groups for the diagnosis of cirrhosis and fibrosis quantification. The degree of liver fibrosis was categorized into five groups according to the following scoring system [21, 22]: 0, no fibrosis, normal liver and absence of fibrosis; I, fibrosis present (collagen fibres present that extend from the portal triad or central vein to the peripheral region); II, mild fibrosis (mild collagen fibres present with extension without compartment formation); III, moderate fibrosis (moderate collagen fibres present with a certain level of pseudo-lobe formation); and IV, severe fibrosis (severe collagen fibres present with thickening of the partial compartments and frequent pseudo-lobe formation).

### Liver function test

Serum was collected at day 21 after cell transplantation. The alanine transaminase (ALT), aspartate transaminase (AST), albumin and total protein were analysed using a semi-automatic biochemical analyser (BT-815A, Shanghai Sanco Instrument Co. Ltd., China).

### Quantitative real-time RT-PCR

Total RNA samples were extracted using Trizol (Invitrogen, CA, USA) according to the manufacturer’s instructions. Real-time quantitative PCR analysis was performed using Applied Biosystems 7500 Real-Time PCR Systems (Applied Biosystems, Foster City, CA). The housekeeping gene GAPDH was used as an internal control for relative quantification. The mRNAs of Smad2, Smad3, Smad7, TGF-β_1_, TGFBR1, TIMP-1, MMP1, collagen I, collagen III and α-SMA were quantified using the SYBR green method after cell transplantation for 7 and 21 days. The real-time PCR procedure and the calculation of relative quantification were similar to our previous reports [23, 24]. The primer sets used in this study were purchased from Invitrogen (Supplementary Table 1).

### Western blotting and ELISA detection

The Western blotting procedure is similar to our previous reports [17, 23]. Briefly, the liver tissue from the rat model was lysed in RIPA buffer containing a proteinase inhibitor cocktail (Biocolor BioScience & Technology, Shanghai, China). The protein concentration was determined using bicinchoninic acid (Bioss, Beijing, China). Protein was loaded at 30 μg/gel each lane, separated on 10% SDS-PAGE and transferred to nitrocellulose membranes. The blots were incubated with rabbit primary antibodies anti-MMP-1 (1:1000, sc-241561) or TIMP-1 (1:1000, sc-5538) (Santa Cruz Biotech, USA) and anti-GAPDH (rabbit monoclonal antibody, 1:1000, Ab181602, Abcam, UK) at 4 °C overnight, then washed extensively with 0.1% Tween-20 in PBS and incubated with a secondary antibody conjugated to horseradish peroxidase (1:5000; sc-2004, Santa Cruz, USA) at room temperature for 3 h. The blot was visualized using the ECL system (Amersham, UK).

The serum levels of Smad7, collagen I, collagen III, laminin and hyaluronic acid were detected using ELISA according to the manufacturer’s instruction. The ELISA kits for Smad7 (# CSB-E09225r) and hyaluronic acid (# CSB-E08120r) were purchased from CUSABIO Technology LLC (Wuhan, China), and the kits for collagenase I (# CX20064), collagenase III (# kt210320) and laminin (# KT20202) were from MSKBIO (Wuhan, China).

### Statistics

Data are expressed as mean ± standard deviation (SD). One-way analysis of variance (ANOVA) was used to compare the means from more than three groups, and unpaired *t* test was used for two group comparison. A value of *P* < 0.05 was considered a significant difference.

## Results

### MSC primary culture and transduction efficiency with the lentivirus vector

The isolated bone marrow MSCs expanded extensively in the culture medium from single cell-derived colonies in ~ 2 weeks under optimal conditions. Morphological examples of isolated MSCs from passage 1 were shown (Supplementary Fig. 1A). The phenotypes of MSCs were characterized by positive and negative cell markers (CD31, CD34, CD45, CD73, CD105 and CD106) using a flow cytometer as we reported previously [17]. After obtaining enriched MSCs, the cells were transduced with the lentivirus vector carrying the EGFP-Smad7 gene. The condition of transduction was optimized by using different MOIs in the range 5–80, and the transduction efficiency was assessed using fluorescence microscopy. The protein expression of EGFP-Smad7 was evident after the removal of the virus supernatant and more than 95% of cells were positive at day 3 or even higher at day 5 (Supplementary Fig. 1B). The MSCs overexpressing Smad7 at passage 3 were used for in vivo cell therapy in the following liver cirrhosis model.

### Establishment of the rat liver cirrhosis model

**Fig. 1:**
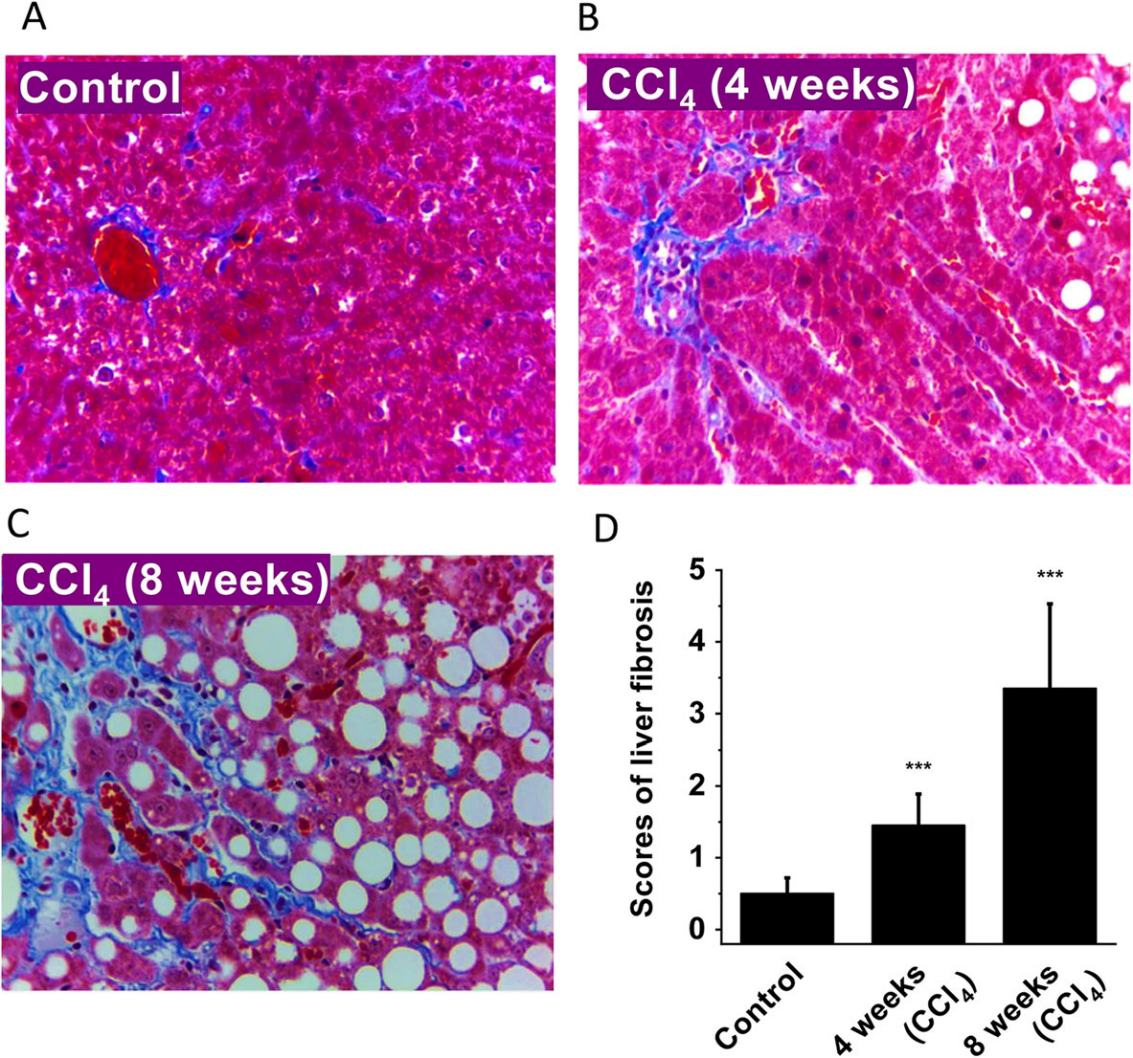
CCl_4_-induced liver fibrosis assessed by Masson’s trichrome staining (× 400). **a** Normal rat liver tissue as control. **b** Rat liver tissue obtained at 4 weeks after injection of CCl_4_ (prior to cirrhosis). **c** Typical liver cirrhosis tissue section after injection of CCl_4_ for 8 weeks. **d** Histological scores for liver fibrosis (*n* = 5 for each group). ***P < 0.001 comparing with the control group

Rat liver cirrhosis was induced by subcutaneously injecting 40% carbon tetrachloride (CCl_4_) in rapeseed oil. The liver fibrosis was evaluated by Masson’s trichrome staining. In normal rat liver tissue sections, the structure of the hepatic lobule was clear and there was no evident collagen fibres present or very few fibres extended from the portal triad or central vein to the peripheral region of the hepatic cords (Fig. 1a). After injection of CCl_4_ for 4 weeks, hepatocellular fatty degeneration and hepatic cell necrosis accompanied with obvious inflammation were present. The increased collagen staining with evident fibres extended from the central vein to the peripheral region, which represented the stage prior to cirrhosis (Fig. 1b). After injection of CCl_4_ for 8 weeks, the liver cirrhosis was evident, which was characterized by disordered architecture of hepatic cords and lobule structure, fibrous tissue and hyperplasia, collagen production, and a large number of inflammatory cells infiltrated in the portal area and surrounding central veins (Fig. 1c). The mean scores of liver fibrosis for normal liver tissue and liver tissues obtained at early (4 weeks) and late (8 weeks) stages of the CCl_4_-induced cirrhosis were 0, 1.45 and 3.35, respectively (Fig. 1d), suggesting the successful induction of rat liver cirrhosis after injection of CCl_4_.

### Enhanced expression of Smads7 after Smad7-MSC therapy

**Fig. 2:**
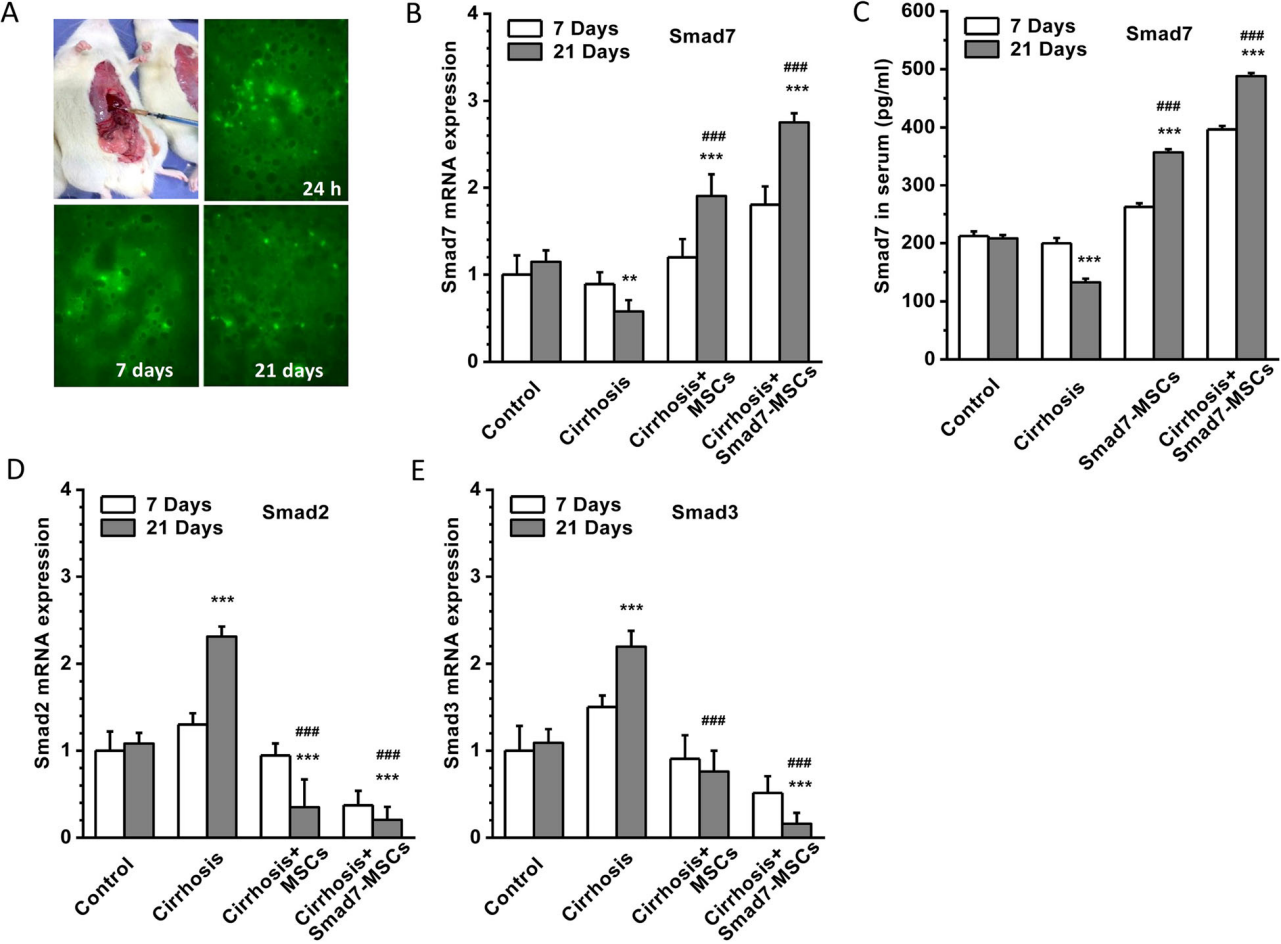
Distribution of Smad7-EGFP-MSCs in the liver and the expression of Smads. **a** Example of direct injection of Smad7-EGFP-MSCs into the liver lobes. The distribution of Smad7-EGFP-MSCs (green fluorescence) in the liver tissue was detected on the frozen sections using a laser confocal microscope at the time points of 24 h, 7 days and 21 days after cell injection. **b** Smad7 mRNA quantification in the groups of control (vehicle control without CCl_4_), cirrhosis group (with CCl_4_ injection), cirrhosis group treated with MSCs and cirrhosis group treated with Smad7-MSCs. **c** Serum protein level of Smad7 detected using ELISA (*n* = 4). **d** Smad2 mRNA quantification using real-time PCR. GAPDH was set as housekeeping gene control for relative quantification (*n* = 6). **e** Smad3 mRNA quantification (*n* = 6). **P < 0.01 or ***P < 0.001 comparing with the control group; ^###^*P* < 0.001 comparing with the cirrhosis group

In order to confirm if the Smad7-EGFP-MSCs were successfully transplanted into the liver, one rat from each group was sacrificed at 24 h, 1 week and 4 weeks after cell transplantation and the livers were dissected out for frozen sectioning. The distribution of Smad7-EGFP-MSCs was examined under a confocal microscope, and MSCs with green fluorescence were diffused at the portal area after 24-h cell transplantation. After cell transplantation for 7 days, the cells with green fluorescence were migrated and appeared in the necrotic area of the liver, and after 21 days of transplantation, the Smad7-MSCs were more sparsely distributed in the liver (Fig. 2a). This suggested that the transplanted Smad7-EGFP-MSCs can survive in the liver and migrate in the live lobules.

The expression of Smad7 in the liver tissue was detected by real-time PCR. The Smad7 mRNA was decreased in the CCl_4_-induced liver cirrhosis group. After treatment with Smad7-MSCs, the expression of Smad7 was significantly increased in the recipient liver with cirrhosis. Smad7 was also increased in the group treated with MSCs alone compared to the control. The enhanced expression of Smad7 was more robust after the cell transplantation for 21 days (Fig. 2b). The serum level of Smad7 protein was also determined using ELISA, and the Smad7 was significantly increased after transplantation of Smad7-MSCs or MSCs. The Smad7 levels for the group treated with Smad7-MSCs were much higher than those for the other groups (Fig. 2c).

Smad2 and Smad3 as effectors of TGF-β signalling were also detected in the liver tissue using real-time PCR. The enhanced expression of Smad2 and Smad3 was observed after the induction of liver cirrhosis. After cell transplantation, the expression levels of both Smad2 and Smad3 were decreased (Fig. 2d, e), suggesting that enhanced Smad7 activity after cell therapy may affect other SMAD isoform expression.

### Effects on fibrosis biomarkers

**Fig. 3:**
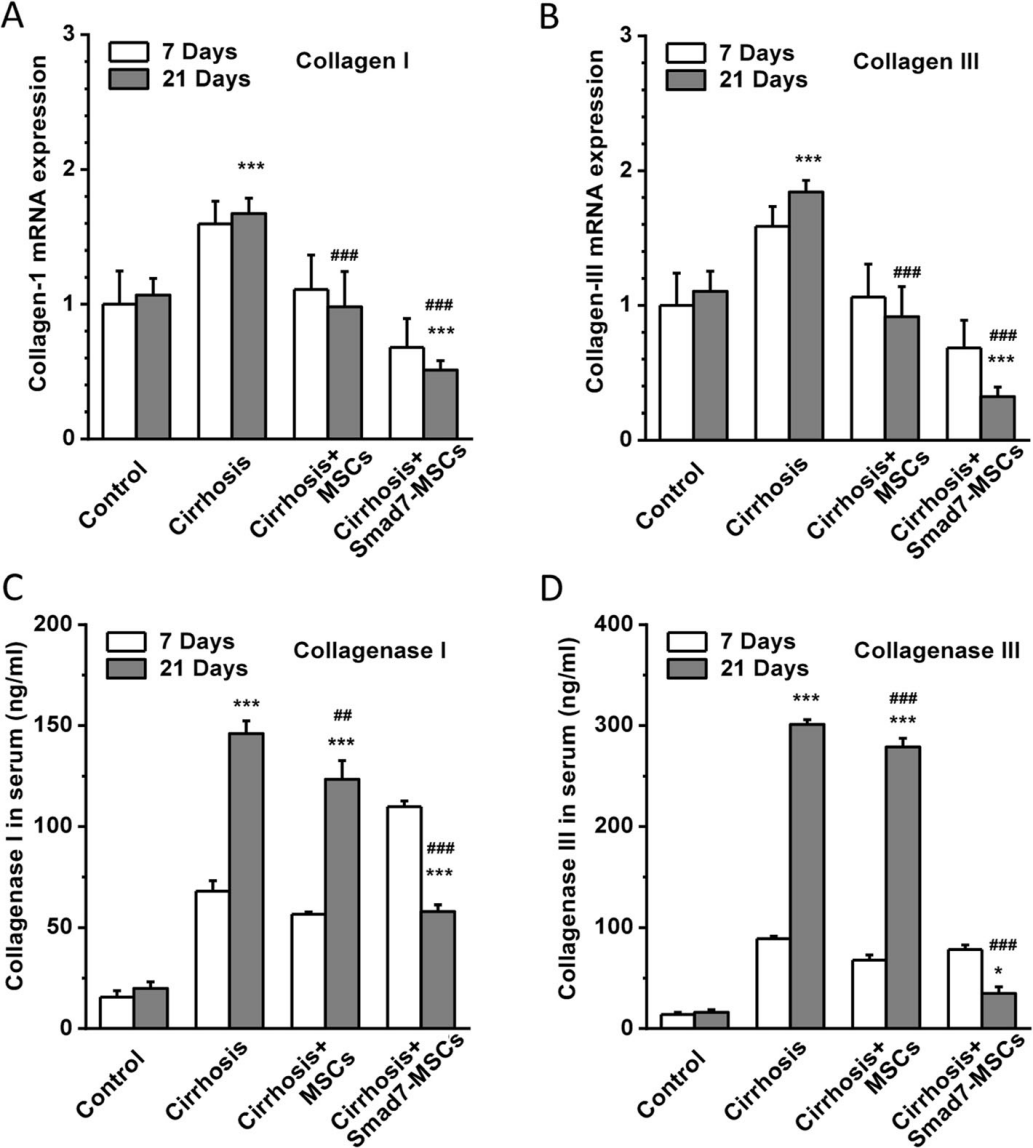
Detection of fibrosis biomarkers collagen and collagenase. **a** Real-time PCR detection of collagen I and collagen III expression in the liver tissues treated with Smad7-MSCs, comparing with the control (vehicle), cirrhosis group (with CCl_4_ injection) and the cirrhosis group treated with MSCs (*n* = 6 for each group). **b** Quantification of collagen III mRNA (*n* = 6 for each group). **c** ELISA detection of collagenase 1 and collagenase III (**d**) in rat serum (*n* = 4 for each group). *P < 0.05 or ***P < 0.001 comparing with the control group; ^##^*P* < 0.01 or ^###^*P* < 0.001 comparing with the cirrhosis group

The mRNAs of collagen I and III were detected using real-time PCR. After induction of liver cirrhosis with CCl_4_ injection, the collagen I and III were significantly increased in comparison to the control group. After transplantation with MSCs, the enhanced expression of collagen mRNAs was reduced, and the reduction in the group treated with Smad7-MSCs for 21 days was much more significant (Fig. 3a, b).

The serum collagenase I and III were detected using ELISA. The peripheral blood was collected, and the serum was isolated from the rats after MSC transplantation for 7 days and 21 days. The levels of collagenase I and III were significantly increased in the CCl_4_-induced liver cirrhosis group. Treatment with Smad7-MSCs significantly reduced the level of collagenases (Fig. 3c, d), suggesting that the cell therapy with Smad7-MSCs is effective in the prevention of liver fibrosis.

### Effects on TGF-β1, TGFBR1 and α-SMA

**Fig. 4:**
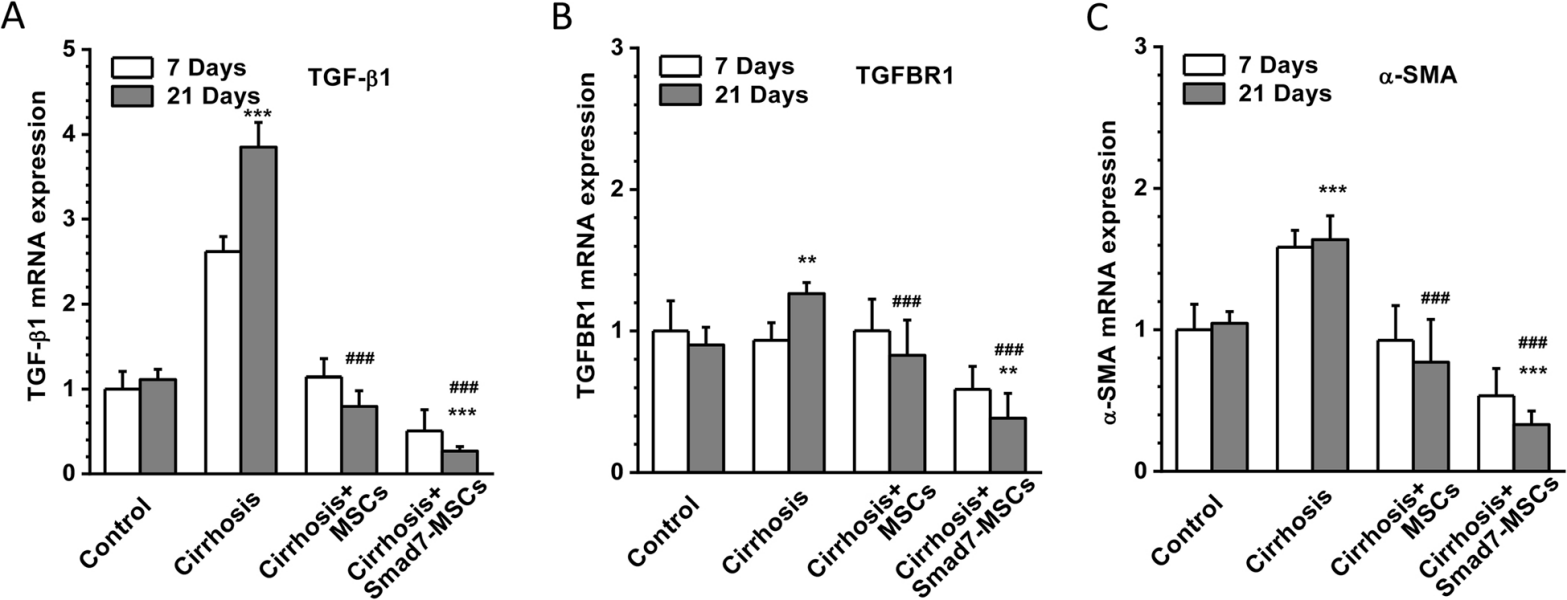
Downregulation of TGF-β1, TGFBR1 and α-SMA after Smad7-MSC treatment. Real-time PCR detection of TGF-β1 (**a**), TGFBR1 (**b**) and α-SMA (**c**) expression in the rat liver tissues treated with Smad7-MSCs, comparing with the control (vehicle), cirrhosis group (with CCl_4_ injection) and the cirrhosis group treated with MSCs (*n* = 6 for each group). The GAPDH was set as the housekeeping gene for relative quantification (*n* = 6 for each group). **P < 0.01 or ***P < 0.001 comparing with the control group; ^###^*P* < 0.001 comparing with the cirrhosis group

The mRNAs of TGF-β1, TGFBR1 and α-SMA were quantified using real-time PCR. Induction of liver cirrhosis with CCl_4_ significantly increased the expression of TGF-β1 and α-SMA; however, TGFBR1 expression was not significantly changed. After transplantation of Smad7-MSCs, the expression of TGF-β1 and TGFBR1 (Fig. 4a, b) was decreased, suggesting the potential alleviation of TGF-β1 signalling activity after Smad-7 cell therapy. The α-SMA is a biomarker of myofibroblasts and also acts as an indicator of HSC activation, which precedes fibrous tissue deposition in the liver [25]. The expression of α-SMA was increased in the CCl_4_-induced liver cirrhosis group; however, the expression was significantly decreased in the group receiving Smad7-MSCs (Fig. 4c), suggesting less activity of HSCs occurred in the liver after cell therapy.

### Effects on laminin, hyaluronic acid, MMP-1 and TIMP-1

**Fig. 5:**
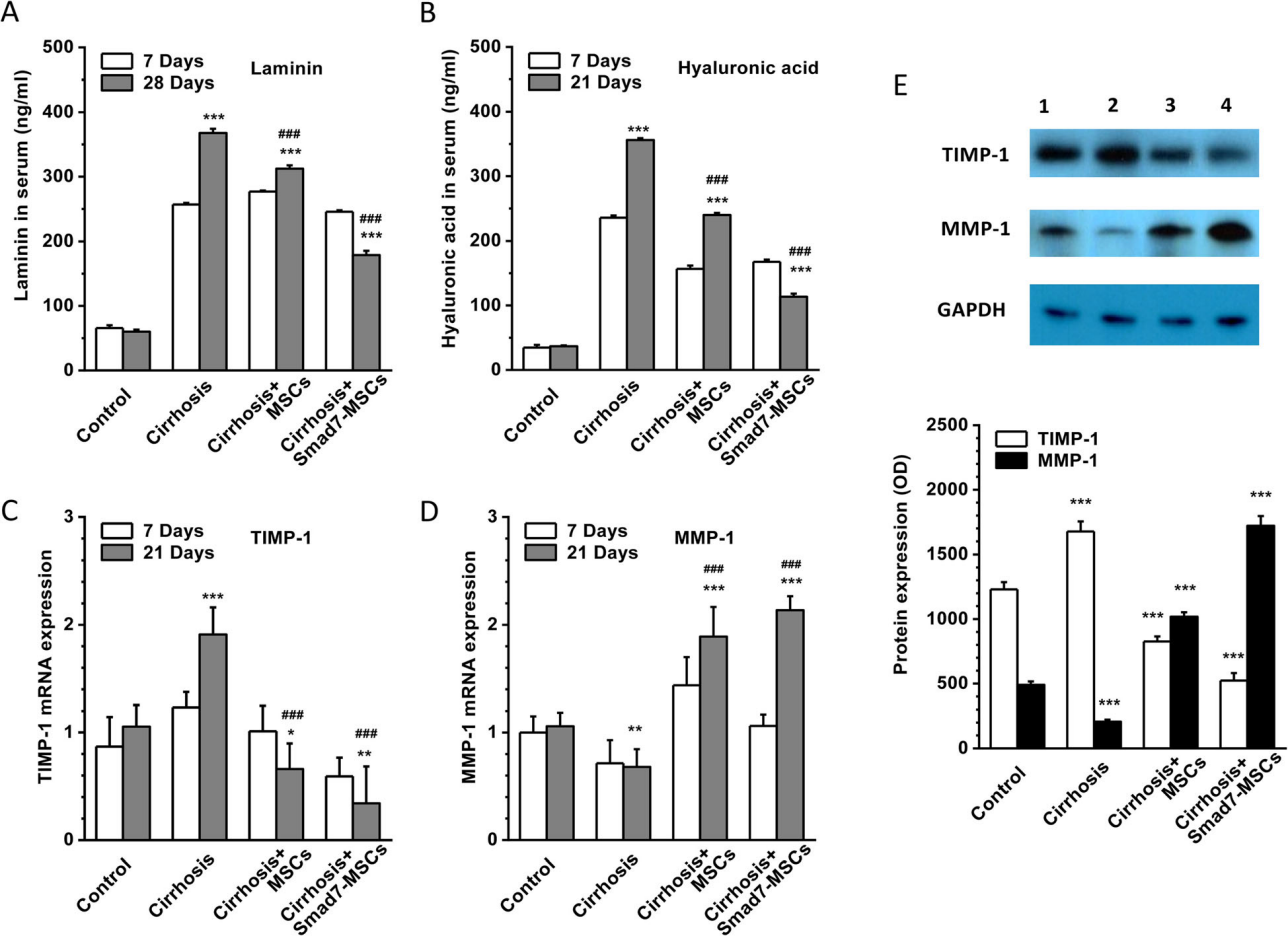
Changes in extracellular matrix protein laminin, hyaluronic acid, TIMP-1 and MMP-1. ELISA detection of laminin (**a**) and hyaluronic acid (**b**) in the sera from the rats treated with Smad7-MSCs for 7 and 21 days in comparison with the control group (vehicle), cirrhosis group (with CCl_4_ injection) and the cirrhosis group treated with MSCs (*n* = 4 for each group). **c**, **d** Quantitative PCR for TIMP1 and MMP-1. GAPDH was used as the housekeeping gene for relative quantification (*n* = 6 for each group). **e** TIMP-1 and MMP-1 detected by Western blotting in the liver lysate (*n* = 5 for each group). *P < 0.05, **P < 0.01 or ***P < 0.001 comparing with the control group; ^###^*P* < 0.001 comparing with the cirrhosis group

The serum level of laminin and hyaluronic acid was measured using ELISA after MSC transplantation for 7 days and 21 days. The serum levels of laminin and hyaluronic acid were significantly increased in the CCl_4_-induced liver cirrhosis group. Treatment with Smad7-MSCs reduced the serum levels of the two proteins (Fig. 5a, b), suggesting that the cell therapy with Smad7-MSCs can change the extracellular matrix proteins.

MMP-1 is a member of the MMP family, which is responsible for the type I and type III collagen degradation in ECM. TIMP-1 is an active polypeptide that can inhibit the function of MMP-1 by forming a reversible covalent complex with MMP-1. The levels of TIMP-1 were positively correlated with the level of liver inflammation and fibrosis, and the levels of MMP-1 were negatively correlated with the level of fibrosis [26]. Our data showed that the mRNA and protein levels of TIMP-1 were remarkedly increased and the levels of MMP-1 were significantly decreased in the CCl_4_-induced cirrhosis liver group. After treatment with Smad7-MSCs for 21 days, TIMP-1 was decreased, while MMP-1 was increased (Fig. 5c–e). These results suggested that the alleviation of liver fibrosis after treatment with Smad7-MSCs could be mediated by the alternation of TIMP-1 and MMP-1 expression.

### Liver fibrosis and liver function improved by Smad7-MSC-base gene therapy

**Fig. 6:**
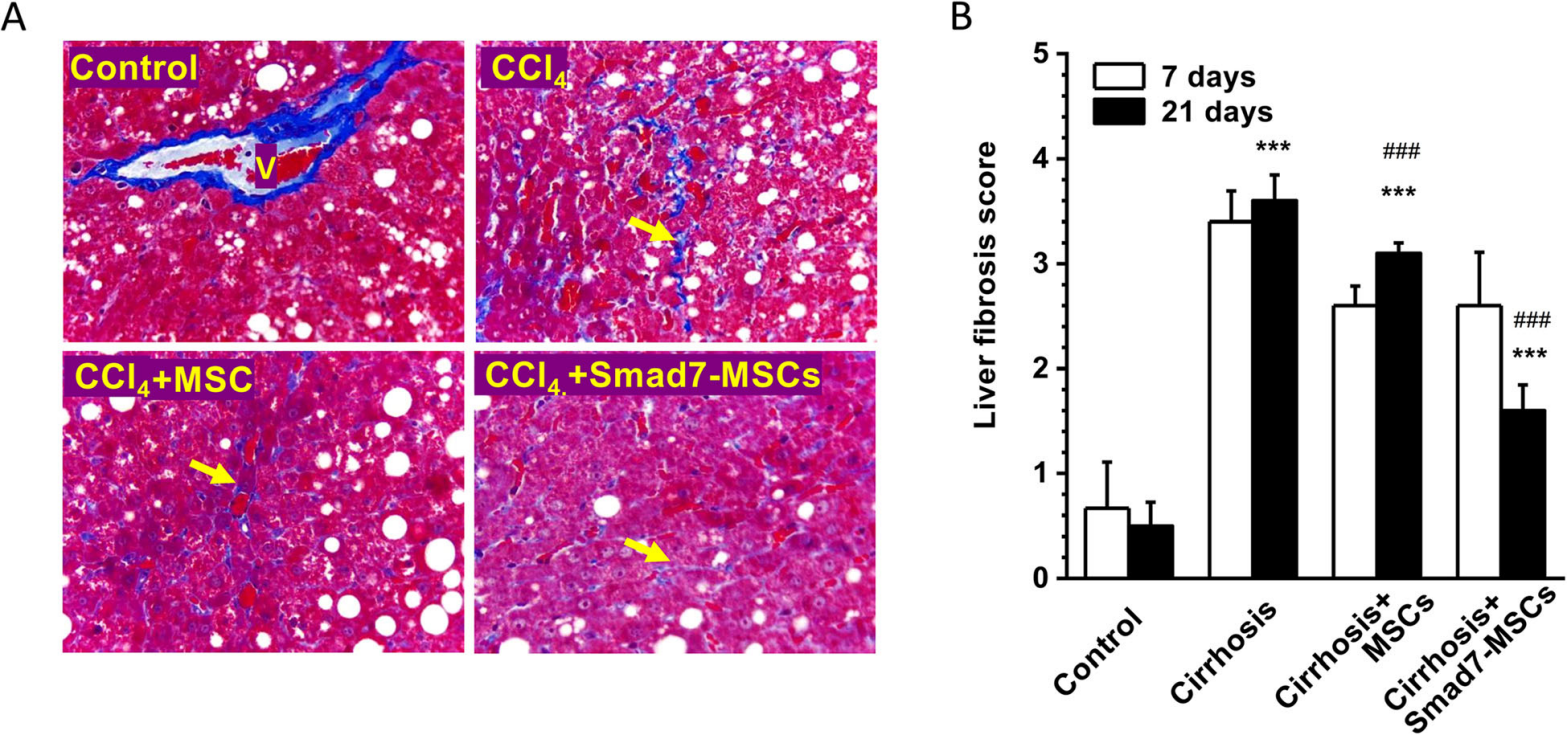
Histopathological evaluation of liver fibrosis after Smad7-MSC therapy. **a** The rat liver tissue sections were stained using Masson’s trichrome stain method and assessed by fibrosis semi-quantitative scoring method (grades 0–5). The isolated collagen fibres (indicated by arrow) and collagen around the wall of central vein (V) were stained blue. Examples for the control group (vehicle), cirrhosis group (with CCl_4_ injection) and the cirrhosis groups treated with MSCs or Smad7-MSCs for 21 days. **b** Mean ± SD for the scoring on severity of fibrosis (*n* = 5 for each group)

In the PBS-treated liver cirrhosis group, the liver fibrosis score achieved 3.6 after initial injection of CCl_4_ for 11 weeks (8-week liver cirrhosis induction + 21-day treatment) and significantly higher than the control group without injection of CCl_4_ (3.6 ± 0.24 vs 0.5 ± 0.22, *n* = 5). Transplantation of MSCs showed a tendency of fibrosis reduction, but did not achieve a statistical difference. However, the group treated with Smad7-MSCs for 21 days significantly reduced histopathological fibrosis scores (Fig. 6), suggesting that Smad7-MSC-base gene therapy can reduce liver fibrosis. The activity of ALT, AST, albumin and total protein were determined after the treatment with Smad7-MSCs for 21 days (Table 1). The ALT and AST were significantly reduced while the total protein level increased comparing with the CCl_4_-treated group, suggesting that the liver function was alleviated.

**Table 1:** Liver function tests for the groups treated with or without CCl_4_ or MSC-Smad7 for 21 days

Group	Number	ALT(U/L)	AST(U/L)	ALB (g/dl)	TP (g/dl)
Control	6	26.8 ± 9.2	66.9 ± 7.8	43.7 ± 9.1	70.7 ± 7.4
CCl_4_-treated	6	128.6 ± 10.3***	138.4 + 6.1***	23.4 ± 8.6**	43.8 + 6.4***
CCl_4_+MSC	6	119.6 ± 10.1***	135.7 ± 6.8***	22.8 ± 8.1**	44.9 ± 6.3***
CCl_4_+MSC-Smad7	6	65.3 ± 11.9*^, ###^	82.9 ± 7.7**^,###^	31.7 ± 7.1*	63.6 ± 5.3^###^

*ALT* alanine transaminase, *AST* aspartate transaminase, *ALB* albumin, *TP* total protein. *P < 0.05, **P < 0.01 and ***P < 0.001 comparing with the control group. ^#^*P* < 0.05, ^##^*P* < 0.01 and ^###^*P* < 0.001 comparing with the CCl_4_-treated group

## Discussion

In this study, we have shown that MSCs overexpressing the Smad7 gene can exert therapeutic effects via the reduction of fibrosis biomarkers (collagen and collagenase activity) and inflammatory markers (TGF-β1 and TGFBR1). The extracellular matrix biomarkers, histopathological score for liver fibrosis, and liver function were improved after MSC-Smad7 cell therapy. Our findings provide a direct in vivo evidence that Smad7-MSC therapy is effective in the treatment of CCl_4_-induced liver cirrhosis.

To restore liver functionality in patients with liver cirrhosis, cell therapy strategies using bone marrow-derived stem cells [6, 27] or MSCs carrying specific genes are sought-after research areas [5, 9]. The treatment with MSCs alone showed some degree of alleviation of liver fibrosis and also inhibited HSC proliferation, which is in accordance with the studies in mouse or rat models [10, 28, 29]. Here we have firstly validated the effectiveness of cell therapy using MSCs overexpressing the Smad7 gene on the CCl_4_-induced in vivo animal model. As we expected, the therapeutic effect was more significant in the group treated with Smad7-MSCs comparing with the group treated with MSCs alone, suggesting that MSCs overexpressing Smad7 are more effective in the treatment of liver cirrhosis via inhibiting TGF-β1-Smad signalling pathway. The expression of Smad2 and Smad3 was also examined in the study; both enhanced expression of Smad2 and Smad3 in the CCl_4_-induced liver cirrhosis rats was decreased after injection with Smad7-MSC or MSCs, suggesting that MSC therapy may affect other gene expression as well and thus exert overall protective effects.

The MSCs isolated from the bone marrow are characterized by physical, morphological, phenotypical and functional properties [30]. The expression of cell markers has been confirmed as previously described [17, 19]. MSCs at passage 3 were used for cell therapy to avoid potential phenotype change. The migration of Smad7-EGFP-MSCs in the liver was examined after direct injection into different lobes via the detection of the fluorescent report protein, EGFP. The underlying mechanisms for MSCs in the treatment of liver cirrhosis are still unclear [31]. The explanations could be as follows: (1) MSCs may proliferate and differentiate into liver cells in the liver microenvironment. The enhanced levels of Smad7 in the liver and in the serum suggest the possibility of cell number expansion in the body after cell transplantation. (2) MSCs have been shown anti-inflammatory and thus can reduce the activation of HSCs [32]. The decreased expression of inflammatory biomarkers was also observed in this study after Smad7-MSC or MSC treatment. (3) MSCs have been recently reported to affect macrophage function, which will affect immune response and tissue repair in the damaged liver, (4) MSCs change matrix metalloproteinases to reduce the ECM. Our results on MMP1 and TIMP1 support this hypothesis. (5) The paracrine/endocrine mechanisms may exist via the up- or downregulation of inflammatory factors or cytokines after receiving MSCs [33]. (6) The exosome of MSCs and cell culture media only seem to have an effect, suggesting that the mechanism is not due to the cell itself, but due to the secretion or humoral factors released from the cells [9].

Due to the high cost, scarce source of donors and other limitations, liver transplantation is still very limited in clinical practice, and artificial liver support systems can only temporarily replace and assist liver function, but they are unable to reduce the mortality of patients with liver cirrhosis. Stem cells have low immunogenicity and high potency for proliferation and differentiation, which may play a key role in the treatment of liver cirrhosis. Organ injection of MSCs is a feasible approach in clinical settings. The MSCs which overexpress a specific gene, such as Smad7 in this study, may achieve maximum clinical efficacy via targeting specific signalling pathways, which may shed a light on future therapeutics for end-stage liver disease. In addition, targeting TGF-β1-Smad7 signalling is applicable to the fibrosis of other organs, such as lung fibrosis [34], and thus, MSC-Smad7 cell therapy should open a new strategy for anti-fibrosis therapy.

## Conclusions

In this study, MSC-Smad7 cells were transplanted into the liver of rats with CCl_4_-induced liver cirrhosis and the inflammatory and fibrosis biomarkers in serum and liver tissue were determined. The liver fibrosis biomarkers and histology score were significantly mitigated after stem cell transplantation. The findings from this in vivo rat model provide the first direct in vivo evidence to suggest that the MSC-Smad7 cell therapy is an effective approach to prevent liver cirrhosis development, which is of clinical significance for future therapeutic development.

## Supplementary information

**Additional file 1: Supplementary Fig. 1**. Example of cultured bone marrow MSCs and the expression of Smad7-EGFP. (A) Example of MSCs isolated from rat bone marrow and cultured at passage 1. (B) MSCs infected with a lentiviral vector carrying Smad7-EGFP gene. **Supplementary Fig. 2**. Examples of western blotting. The cropped box areas are presented in Fig. 5. Lane 1: Cirrhosis+MSC-Smad7 (21 day); Lane 2: Cirrhosis+BMSCs (21 day); Lane 3 Cirrhosis (21 day); Lane 4: Conrol (21 day); Lane 5: Cirrhosis+MSC-Smad7 (7 day); Lane 6: Cirrhosis+BMSCs (7 day); Lane 7: Cirrhosis (7 day); Lane 8: Control (7 day). **Supplementary Table 1**. Primer sequences for RT-PCR.

## Data Availability

All data generated and/or analysed during this study are included in this published article. Data sharing is not applicable to this article as no datasets were generated or analysed during the current study. However, the data that support the findings of this study are available from the corresponding author upon reasonable request.

## Abbreviations

α-SMA: Alpha-smooth muscle actin
ALT: Alanine transaminase
AST: Aspartate transaminase
CCl_4_: Carbon tetrachloride
ECM: Extracellular matrix
HBV: Hepatitis B virus
HSCs: Hepatic stellate cells
MMP-1: Matrix metalloproteinase 1
MSCs: Mesenchymal stem cells
Smad7: Mothers against decapentaplegic homolog 7
TGF-β1: Transforming growth factor-β1
TGFBR1: TGF beta 1 receptor 1
TIMP-1: Tissue inhibitors of metalloproteinase-1
