# Supplementary material for: Mesenchymal stem cell-based Smad7 gene therapy for experimental liver cirrhosis
Source: Stem Cell Res Ther. 2020 Sep 14;11:395. doi: 10.1186/s13287-020-01911-4 (PMC7489041; doi:10.1186/s13287-020-01911-4)
Supplement: Supplementary file 1 — Additional file 1: Supplementary Fig. 1. Example of cultured bone marrow MSCs and the expression of Smad7-EGFP. (A) Example of MSCs isolated from rat bone marrow and cultured at passage 1. (B) MSCs infected with a lentiviral vector carrying Smad7-EGFP gene. Supplementary Fig. 2. Examples of western blotting. The cropped box areas are presented in Fig. 5. Lane 1: Cirrhosis+MSC-Smad7 (21 day); Lane 2: Cirrhosis+BMSCs (21 day); Lane 3 Cirrhosis (21 day); Lane 4: Conrol (21 day); Lane 5: Cirrhosis+MSC-Smad7 (7 day); Lane 6: Cirrhosis+BMSCs (7 day); Lane 7: Cirrhosis (7 day); Lane 8: Control (7 day). Supplementary Table 1. Primer sequences for RT-PCR. [file 13287_2020_1911_MOESM1_ESM.pdf]

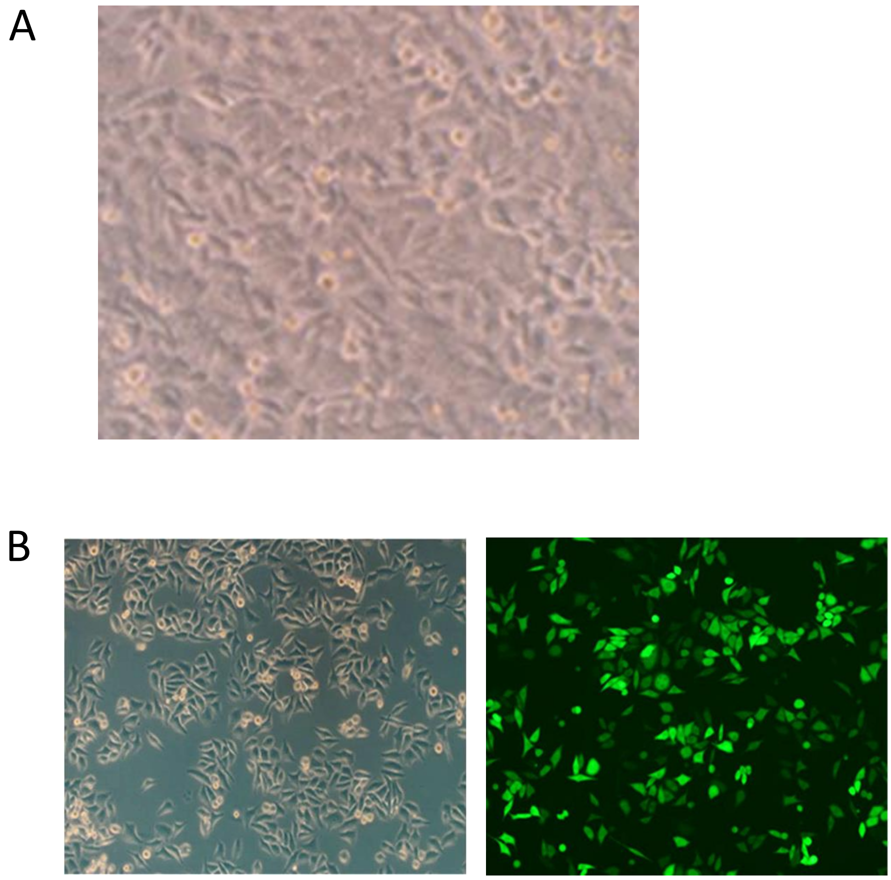

Supplementary Figure 1. Example of cultured bone marrow MSCs and the expression of Smad7-EGFP. (A) Example of MSCs isolated from rat bone marrow and cultured at passage 1. (B) MSCs infected with a lentiviral vector carrying Smad7-EGFP gene.

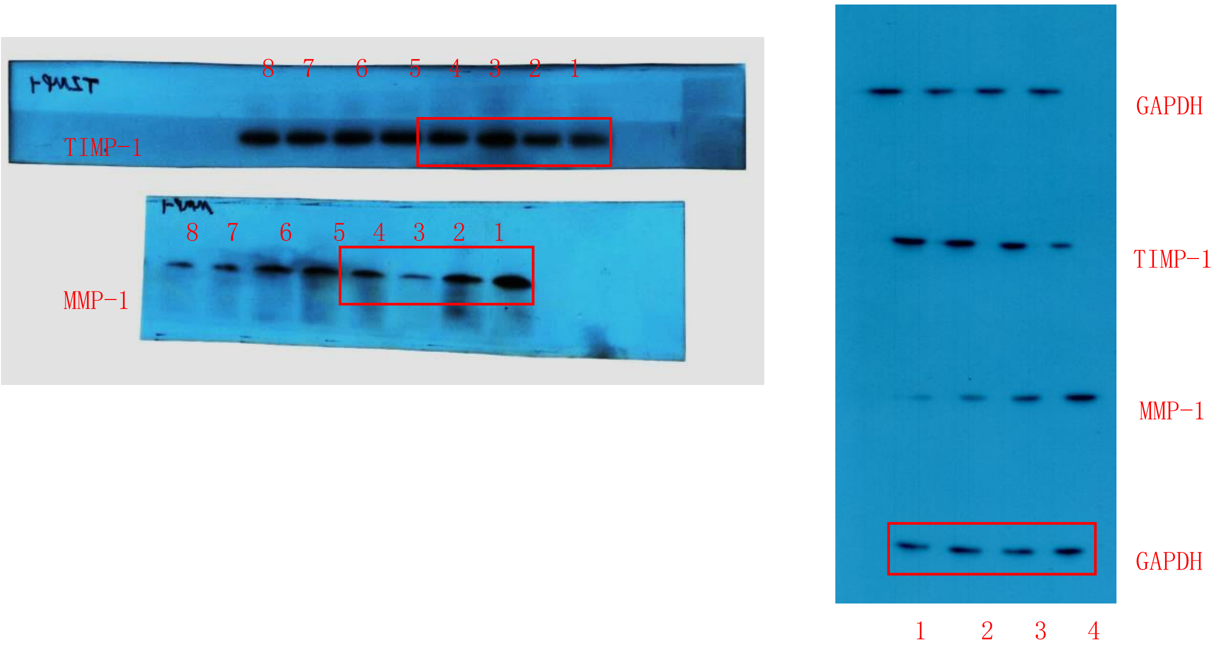

Supplementary Figure 2. Examples of western blotting. The cropped box areas are presented in Figure 5. Lane 1：Cirrhosis+MSC-Smad7 (21 day); Lane 2: Cirrhosis+BMSCs (21 day); Lane 3: Cirrhosis (21 day); Lane 4：Control (21day); Lane 5：Cirrhosis+MSC-Smad7 (7 day); Lane 6: Cirrhosis+BMSCs (7 day); Lane 7: Cirrhosis (7 day); Lane 8：Control (7 day).

Supplementary Table 1. Primer sequences for RT-PCR

**Gene**	**Accession No**	**F/R**	**Primer sequence (5' to 3')**	**Location**	**Size**
Smad7	AH008243.2	F	5'-GGGTTTACA ACCGCAGCAGT-3'	4032-4051	112
		R	5'-GCCTTGATGGAGAAACCAGG-3'	4143-4124	
TGFβ1	NM_021578.2	F	5'-AAACGGAAGCGCATCGAA-3'	269-286	69
		R	5'-GGGACTGGCGAGCCTTAGTT-3'	331-312	
Smad2	BC127497.1	F	5'-GTGTTTGCCGAGTGCCTAAGT-3'	1114-1134	110
		R	5'-TTACAGCCTGGTGGGATTTTG-3'	1223-1203	
Smad3	NM_013095.3	F	5'-GGACGCAGGCTCTCCAAAC-3'	802-820	110
		R	5'-AGGAGATGGAGCACCAAAAGG-3'	911-891	
TIMP-1	AY550026.1	F	5'-GATTCGACGCTGTGGGAAAT-3'	236-255	107
		R	5'-AAACTCCTCGCTGCGGTTCT-3'	342-323	
Collagenase1	BC133728.1	F	5'-CTCCCAGCGGTGGTTATGAC-3'	3634-3653	151
		R	5'-TGCTGGCTCAGGCTCTTGA-3'	3784-3766	
Collagenase3	NM_032085.1	F	5'-CCCTCTCTTATTTTGGCACAGC-3'	156-177	104
		R	5'-TGACATGGTTCTGGCTTCCAG-3'	259-239	
a-SMA	BC158550.1	F	5'-AATGGCTCCGGGCTCTGTA-3'	91-109	106
		R	5'-TTCCAACCATCACTCCCTGG-3'	196-177	
TGFBR1	BC087035.1	F	5'-GCTGTGGTTGGTGTCGGATT-3'	831-850	103
		R	5'-CGTGGACAGAGCGAGTTTGAT-3'	933-913	
MMP-1	NM_001134530.1	F	5'-CACAACAATCCTCGTTGGACC-3'	343-363	104
		R	5'-AAGGCTCTCTCGATGGCGTT-3'	446-427	
GAPDH	NM_017008.4	F	5'-AGGGCTGCCTTCTCTTGTGA-3'	127-147	110
		R	5'-AACTTGCCGTGGGTAGAGTCA-3'	236-216	
